# Twenty-fifth Annual Report of the Managers of the New York Asylum for Lying-in-women

**Published:** 1848-07

**Authors:** 


*Twenty-fifth Annual Report of the Managers of the New York Asylum for Lying-in-Women.* Presented March 22, 1848.

This charitable institution has been in existence a quarter of a century, in which time it has undoubtedly done much to relieve the sufferings of many unhappy women. We do not discover from the present Report whether the objects of the charity are married women exclusively, or whether its benevolence is more expanded.

It appears that ninety-five patients were admitted during the last year, two of whom, however, did not prove to be proper objects of the charity. The majority of the cases of labour are reported to have been natural—five having been deviations. In the out-door department there were 342 applicants.

We are pleased to observe that the Managers award proper credit for ability and faithfulness to their "excellent Physician," Dr. Stimson, who has the immediate charge of the patients in the Asylum, as well as to the "district physicians," of which there are eighteen.

A proper acknowledgment of such important services by *kind words*, is in general all that physicians get in public institutions of this kind, and too frequently even that poor pay is withheld.

